# The Influence of Bacteria Causing Subclinical Mastitis on the Structure of the Cow’s Milk Microbiome

**DOI:** 10.3390/molecules27061829

**Published:** 2022-03-11

**Authors:** Łukasz Kaczorowski, Jolanta Powierska-Czarny, Łukasz Wolko, Agnieszka Piotrowska-Cyplik, Paweł Cyplik, Jakub Czarny

**Affiliations:** 1 Institute of Forensic Genetics, Al. Mickiewicza 3/4, 85-071 Bydgoszcz, Poland; l.kaczorowski@igs.org.pl (Ł.K.); igs@igs.org.pl (J.P.-C.); 2 Department of Biochemistry and Biotechnology, Poznan University of Life Sciences, Dojazd 11, 60-632 Poznań, Poland; lukasz.wolko@up.poznan.pl; 3 Department of Food Technology of Plant Origin, Poznan University of Life Sciences, Wojska Polskiego 31, 60-624 Poznań, Poland; agnieszka.piotrowska-cyplik@up.poznan.pl; 4 Department Biotechnology and Food Microbiology, Poznan University of Life Sciences, Wojska Polskiego 48, 60-627 Poznań, Poland; pawel.cyplik@up.poznan.pl

**Keywords:** lactic acid bacteria, mastitis, microbiome, milk

## Abstract

Mastitis is the most expensive disease of dairy cattle across the world and is the main reason for the use of antibiotics in animal husbandry. The aim of this study was to analyze the microbiome of raw milk obtained from a semi-subsistence farm located in the Kuyavian–Pomeranian Voivodeship in Poland. Milk from healthy cows and from cows with subclinical mastitis was analyzed. The following pathogenic bacteria were found in milk from individuals with subclinical mastitis: *Escherichia coli* or *Streptococcus agalactiae*. The composition of drinking milk was assessed on the basis of 16S rRNA gene sequencing using the Ion Torrent platform. Based on the conducted research, significant changes in the composition of the milk microbiome were found depending on the physiological state of the cows. The microbiome of milk from healthy cows differed significantly from the milk from cows with subclinical mastitis. Two phyla dominated in the milk from healthy cows: Firmicutes and Proteobacteria, in equal amounts. On the contrary, in the milk from cows with diagnosed subclinical mastitis, one of the types dominated: either Firmicutes or Proteobacteria, and was largely predominant. Moreover, the milk microflora from the ill animals were characterized by lower values of the determined biodiversity indicators than the milk from healthy cows. The presence of pathogenic bacteria in the milk resulted in a significant reduction in the share of lactic acid bacteria in the structure of the population of microorganisms, which are of great importance in the production technology of regional products.

## 1. Introduction

Mastitis is the most common disease of dairy cattle, causing great direct and indirect losses [1,2]. Mastitis is defined as an inflammatory response resulting from the infection of the udder tissues, which can occur in many species of animals kept for milk production [1]. The occurrence of mastitis has a direct impact on the quality of milk, causing changes in both its chemical and physical properties. The most important changes observed in milk are an increase in the number of somatic cells (SSCs), the formation of lumps, or an unfavorable change in color [3]. Initially, only *Streptococcus agalactiae* and *Staphylococcus aureus* were considered as the etiological factors responsible for mastitis; however, further studies have also indicated other species of bacteria, as well as fungi, mycoplasmas, and algae. The most frequently mentioned pathogens, apart from *S. agalactiae* and *S. aureus*, include *Escherichia coli*, *Streptococcus uberis*, *Klebsiella* spp., coagulase-negative staphylococcus (CNS) species, and *Prototheca* spp. [4,5,6].

Direct losses in breeding caused by mastitis include drug and disease management costs, losses of milk that must be discarded, farmer time, mortality among sick animals, and costs associated with the relapse. The shortage of herds, lower animal welfare, and hence, reduced production, as well as a decrease in milk quality, are considered indirect losses [7]. Lower-quality milk is characterized by a shorter shelf life after pasteurization, among other factors, and may also be associated with a lower quality of products such as cheeses obtained using traditional methods (regional products) [3]. Subclinical mastitis is defined as cases when the symptoms are difficult to observe but there is a decrease in the amount of milk produced. The increase in somatic cell count (SSC) is not as pronounced as in clinical mastitis and is most often in the range of 225,000–400,000 cells/mL. Subclinical mastitis leads to slight changes in the properties of milk; however, it may contain pathogens that cause the disease. Due to its often latent course, subclinical mastitis leads to greater losses than clinical mastitis and poses a greater risk of spreading among individuals in a given herd [3,4].

An extremely important aspect of the fight against mastitis is the possibility of carrying out quick, reliable, and precise diagnosis of the disease. The classical methods usually ignore the aspect of mastitis etiology, focusing on symptomatic diagnostics. A lack of targeted treatment, inappropriate selection, and excessive use of antibiotics contribute to the development of drug resistance in bacteria and increase the risk of contamination of milk with trace amounts of antibiotics [8,9].

Meanwhile, domestic animals can be sources of human food poisoning, so to reduce or eliminate this risk, strategies should be developed to prevent animal infections from entering the human food chain [10]. Molecular techniques are one of the most modern technologies in the diagnosis of infectious diseases compared to traditional techniques and have been shown to be much faster and more reliable [11]. These techniques provide a faster and more direct diagnosis of bacterial infections directly from clinical specimens [12].

The development of high-throughput sequencing techniques (including next-generation sequencing) has dramatically reduced the costs and increased the efficiency and accuracy of DNA sequencing, enabling the development of metagenomic or metataxonomic studies in many ecosystems. Molecular approaches such as metataxonomics detect the DNA of all bacteria present in the sample, whether they are alive or not. However, a major limitation of metataxonomic studies is that these approaches describe microbial communities mainly at the genus or higher taxonomic levels, precluding the study of diversity at the species or even strain level [13]. These studies have also provided an understanding of the functional profile of these microbial communities, including data on microbial metabolism, virulence, and antibiotic resistance [14,15]. A review of the studies regarding milk microflora clearly shows the common taxa present in cow’s milk originating from different places. *Staphylococcus*, *Streptococcus*, *Pseudomonas*, *Bifidobacterium*, *Propionibacterium*, *Bacteroides*, *Corynebacterium*, and *Enterococcus* are among the most frequently cited dominant taxa in studies focused on the microbiota of bovine milk [16,17,18]. Moreover, metataxonomic studies have allowed to determine changes in the population of pathogenic bacteria which cause mastitis [19], lactic acid bacteria (LAB) [20], and spoiled milk bacteria [21].

The aim of this study was to determine the influence of *Escherichia coli* and *Streptococcus agalactiae* in milk samples obtained from cows with subclinical mastitis and to determine their influence on the formation of the milk microbiome. The study hypothesis assumed that the presence of microorganisms causing mastitis influences changes in the milk microbiome. In order to limit the influence of factors on the variability of the microbiota, it was decided to analyze the milk from one semi-subsistence farm.

## 2. Results

A total of 55 milk samples from semi-subsistence farms located in the Kuyavian–Pomeranian Voivodeship were analyzed in this study. Determination of SSC was performed in the analyzed samples, which was used to classify the samples into two categories: milk from healthy organisms (24 samples), in which the SSC value was lower than 225,000 cells/mL, and milk with SSC in the range of 225,000–400,000 cells/mL, which corresponds to subclinical mastitis based on veterinary standards [22]. The most common bacteria causing mastitis in cows were detected *(quantitative PCR (qPCR))* in the analyzed samples: *Streptococcus agalactiae* and *Escherichia coli*. In samples marked as H1–H24, with SSC lower than 225,000 cells/mL, the presence of these pathogens was not found, while in samples with SSC values in the range of 225,000–400,000 cells/mL of SSC, both pathogens were identified. Samples in which *S. agalactiae* dominated were marked with symbols from M1 to M15, while those in which *E. coli* was dominant were marked with symbols from E1 to E16 (Table 1). Using the qPCR technique, the abundances of *Escherichia coli* bacteria and *Streptococcus agalactiae* ranged from 10^3^ to 10^5^ cells/mL. Then, all analyzed samples were subjected to taxonomic identification based on the sequence analysis of the hypervariable regions of the 16S rRNA gene based on the SILVA v119 database.

**Figure 1:**
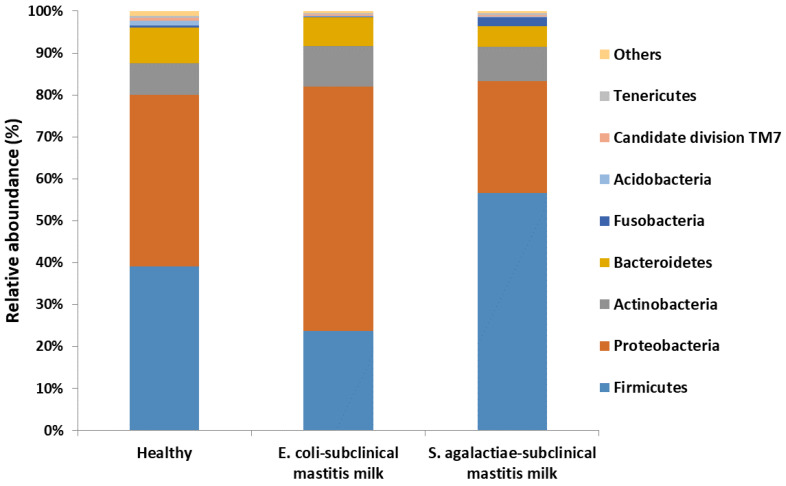
The relative abundance of bacterial phyla in milk from healthy cows and cows with subclinical mastitis.

Two phyla dominated in the samples of milk obtained from healthy cows: Firmicutes and Proteobacteria. Their abundance in the bacterial population was similar and amounted to 39% and 41%, respectively. Other notable phyla present in the milk samples were Actionobacteria (7.5%) and Bacteroidetes (8.5%). The ratio of the remaining phyla of bacteria was lower than 1%. On the contrary, in milk samples with an increased number of SSCs, the relative presence of bacteria changed significantly. Bacteria belonging to Proteobacteria (58.3%) were dominant in the samples in which *E. coli* was found. Meanwhile, the ratio of Firmicutes was equal to 23.7%. In turn, in the samples with the presence of *S. agalactiae*, bacteria belonging to Firmicutes dominated (56.7%), while the ratio of bacteria belonging to Proteobacteria decreased to 26.6% (Figure 1).

**Figure 2:**
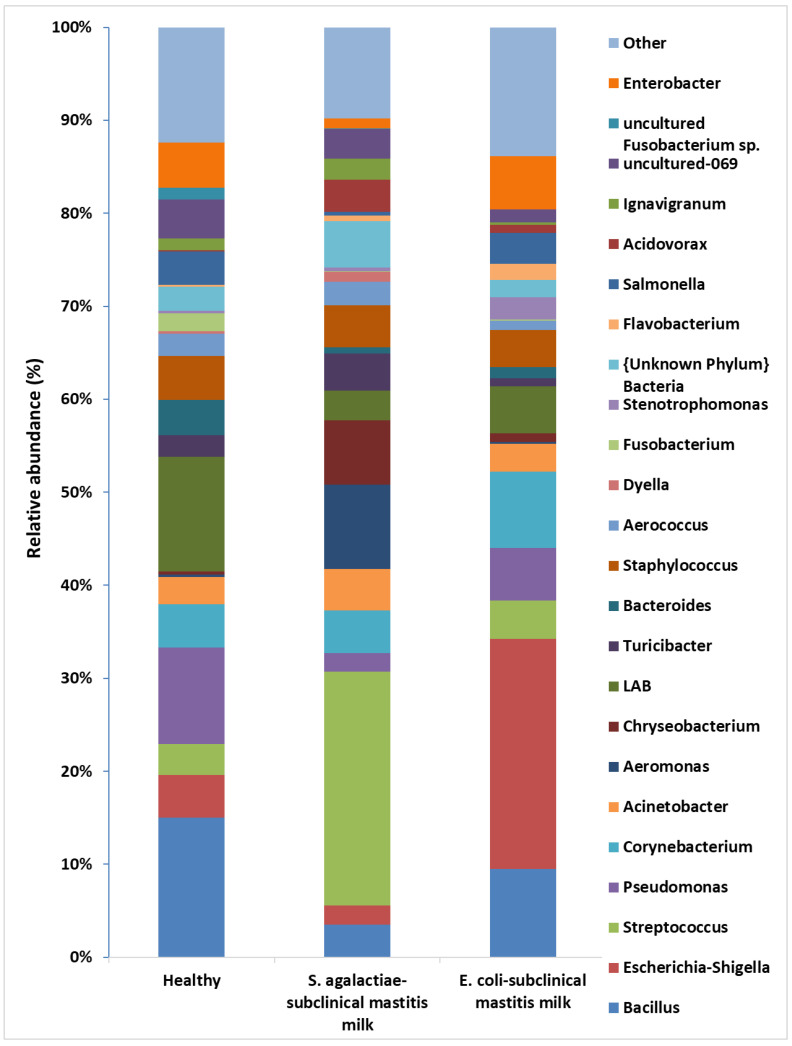
The relative abundance of bacterial genera in the milk from healthy cows and cows with subclinical mastitis.

*Bacillus* dominated in the samples of milk from healthy cows, with an abundance of 15%. The second genus present in milk was *Pseudomonas* (10.3%). The ratio of the other genera did not exceed 5%. On the contrary, in the samples in which *E. coli* bacteria were identified, an increase in the presence of bacteria belonging to the *Escherichia*–*Shigella* genus was visible, the percentage of which was equal to 24.8%. Moreover, a high ratio of bacteria belonging to the *Bacillus* (9.51%) and *Corynebacterium* (8.18%) genera were also observed. However, in the milk samples with the presence of *S. agalactiae* bacteria, a high ratio of *Aeromonas* and *Chryseobacterium* species was also observed, the percentage of which in the population was equal to 9.1% and 6.92%, respectively. In all samples, different amounts of LAB were found, which belonged to the following genera: *Carnobacterium*, *Enterococcus*, *Lacticigenium*, *Lactobacillus*, *Lactococcus*, *Leuconostoc*, *Streptococcus*, and *Trichococcus*. The highest ratio of LAB in the milk microbiome was found in the samples from healthy cows, amounting to 12.32%. However, their presence in the milk from cows with subclinical mastitis was lower and ranged from 3.17% to 5.06% (Figure 2, Table S1).

The values of the analyzed alpha-biodiversity coefficients in the metapopulation of microorganisms present in milk are given in Table 2. Estimates of intrasample diversity were made at a rarefaction depth of 80,000 reads per sample. There was a significant difference in the number of identified operational taxonomic units (OTUs) in all tested trials. In all of the determined indicators, the microbiome of healthy cows was characterized by the greatest biodiversity (Table 2).

**Figure 3:**
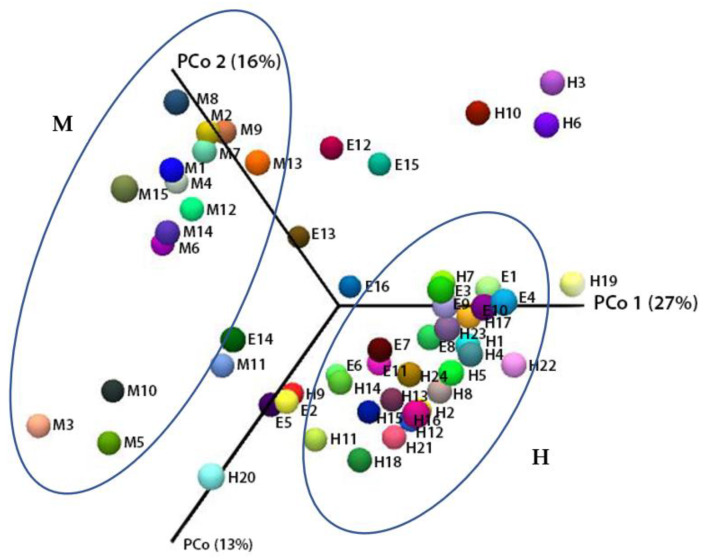
Principal coordinate analysis (PCoA) based on the Bray–Curtis index of bacterial metapopulations in milk (raw data). H1–H24, healthy milk; M1–M15, subclinical mastitis milk with *S. agalactiae*; E1–E16, subclinical mastitis milk with *E. coli*.

Principal coordinate analysis (PCoA) based on the Bray–Curtis index is a measure of the similarity of two populations, based on quantitative and qualitative analysis of the occurrence of each OTU. The figure shows two main areas with clusters marked as H and M. The main place in the chart is taken by points reflecting the microbiome of the milk from healthy cows (H) and the microbiome from subclinical mastitis milk, which is in the second cluster (M) and outside these aggregates. It should also be noted that the variants from healthy (H) and sick (M) cows were significantly different from one another and influenced the metapopulation structure in a different way. The location of points from individual milk samples in two main areas indicates significant differences in the bacterial composition associated with the presence of pathogens disrupting the normal milk microbiome (Figure 3).

**Figure 4:**
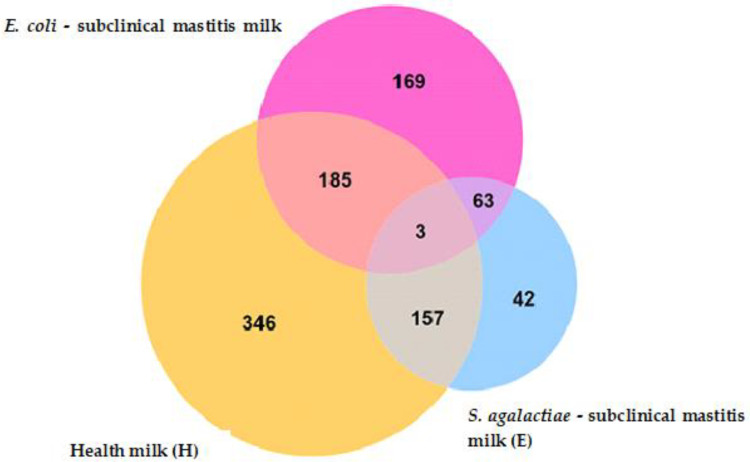
Venn diagram of the microorganisms shared among the milk metagenome. H, healthy milk; M, *S. agalactiae* subclinical mastitis milk; E, *E. coli* subclinical mastitis milk.

Comparative metagenomics of the microbiota of milk from healthy cows and milk from cows with subclinical mastitis (*p* = 0.01) confirmed that the bacteria present in the milk (*S. agalactiae* and *E. coli*) significantly changed the composition of the cow’s milk microbiome. As many as 346 OTUs were unique and only found in cow’s milk. In subclinical mastitis milk, there were 42 unique units for *S. agalactiae*—subclinical mastitis milk (M), while for *E. coli* (subclinical mastitis milk (E)), there were 169. There were 185 taxonomic units common to healthy (H) and *E. coli* (E), while for healthy and *S. agalactiae* (S), there were only 169 taxonomic units. By contrast, only three OTUs were common to all cases. A Venn diagram is shown in Figure 4.

## 3. Discussion

There are many known factors that influence the variability of the raw milk microbiome. These include the hygienic condition of the udder, milking hygiene, the physiological condition of the cows, the number of milking sessions, the method of cleaning and disinfecting equipment, and the type of used feed [23,24]. After analyzing the microbiome of the milk obtained from healthy cows, many authors have confirmed the dominance of bacteria belonging to Firmicutes and Proteobacteria. Most often, the ratio of these phyla is equal to 40–45% [14]. Meanwhile, in milk samples from individuals diagnosed with mastitis, Actinobacteria, Firmicutes, or Proteobacteria dominate, with one of these phyla most often constituting over 70% [19,25].

The composition of the milk microbiome is extremely important because it primarily affects health safety. The composition of the milk microbiome has a significant influence on the pathophysiology of mastitis in cattle. Studies by other authors have confirmed that the diversity of the bacterial microbiome from cattle with subclinical mastitis (E and M) and from healthy cattle (H) results from differences in the abundance of Proteobacteria, Bacteroidetes, Firmicutes, and Actinobacteria [14]. The milk of cows with diagnosed subclinical mastitis is dominated by Proteobacteria, which is the most diverse and includes a wide variety of genera, including *Acinetobacter*, *Pseudomonas*, *Escherichia*, *Vibrio*, *Erwinia*, and *Pantoea*. Firmicutes is dominated by *Streptococcus*, *Enterococcus*, *Staphylococcus*, and *Bacillus*.

On the contrary, in the microbiome of healthy milk, *Acinetobacter*, *Pseudomonas*, *Micromonospora*, *Eubacterium*, *Catenibacterium*, and *Ralstonia* are in greater abundance than in milk from cows with subclinical mastitis. It should also be emphasized that as much as 98% of the total number of microorganisms in healthy cows and cows with diagnosed subclinical mastitis consists of common genera [14].

Family-level taxonomic data showed that the sequences identified from mastitis milk samples with *E. coli* were mainly associated with an increase in the abundance of Enterobacteriaceae, while for *Streptococcus* spp. they were dominated by Streptococcaceae. Similar relationships were described in the work of Lin et al., in which common families accounted for an average relative abundance higher than 85%, regardless of the health status of the milk, the tested milk fraction, or the method of DNA isolation [26].

In the analyzed milk samples (based on phenotypic and genotypic methods), the presence of bacteria belonging to the *Streptococcus agalactiae* species (Group B Streptococcus, GBS) was found. *S. agalactiae* is one of the most common infectious pathogens causing mastitis in dairy cattle. It is a Gram-positive bacterium that often constitutes the normal bacterial flora of humans; however, it can sometimes be a pathogenic microorganism that is particularly dangerous for newborns and the elderly [27]. *S. agalactiae* has the ability to create biofilms, which additionally complicates treatment with antibiotics and extends the viability of bacteria by creating complex three-dimensional structures [28].

*Streptococcus agalactiae* is spread through the udder, mainly from individual to individual, and through contaminated milking equipment [29,30]. Studies performed in China indicate that *S. agalactiae* is the most common etiological factor causing subclinical mastitis [31]. The second group includes environmental pathogens that mainly live in the barn lining [32]. They mostly include opportunistic pathogens, and the infection they cause is usually the result of a weakened animal immunity or physical contact during milking [33].

Environmental pathogens include *Escherichia coli*, which is the most frequently identified Gram-negative bacterium associated with dairy mastitis [34]. It is estimated that up to 40% of clinical mastitis cases can be caused by Gram-negative bacteria, mainly *E. coli* [35]. The symptoms of clinical mastitis, for which *E. coli* is the etiological factor, are very diverse—from local, through mild (red and swollen udders), to severe, extending throughout the body (e.g., fever), which can lead to irreversible changes in tissue and even a complete loss of the ability to produce milk or the death of an individual [35,36].

The studies conducted to date regarding the assessment of the composition of the milk microflora emphasize the importance of LAB lactic acid bacteria, such as *Lactococcus* spp., *Streptococcus* spp., *Lactobacillus* spp., *Leuconostoc*, and *Enterococcus* spp. Raw milk microbiota containing a wide variety of LAB and other microorganisms that are active in human prophylaxis reduce the growth of undesirable microflora in milk, which cause both spoilage of milk and diseases [25]. Apart from lowering the pH, LABs act as competitors of pathogens, as well as produce antimicrobial compounds and lower the carbohydrate content in favor of lactic acid [37].

## 4. Materials and Methods

### 4.1. Characterization of the Farms

The metagenomic analysis was performed for the microflora of milk originating from a farm located in Poland, in the Kuyavian–Pomeranian Voivodeship. The farm was a semi-subsistence farm with seven dairy cows. The samples were collected from June 2020 to July 2021. The cows were of the Holstein Friesian (HF) breed. The working life of the dairy cows was one to three lactations. The average milk yield in the analyzed period was 27.6 kg of milk/animal. Meanwhile, the average number of days of lactation of the cows in a herd was 154–187 days for each month.

### 4.2. Sampling

Samples were collected based on routine procedures on these farm animals. Milk samples were taken after the usual premilking udder preparation by the farmer or milking staff, and before attachment of the milking unit at the same time of day (early in the morning). The teat ends were cleaned with an alcohol swab, the first few strips of milk were discarded, and a sample from all milking quarters was collected into one vial per cow. A clinical examination of the cows was not performed. The milk was collected into 50 mL falcons containing a Broad Spectrum Microtabs II tablet (Bentley Instruments, Chaska, MN, USA). Each tablet, weighing 18 mg, contained 8 mg of bronopol and 0.3 mg of natamycin. Bronopol is a biocide with antibacterial properties, while natamycin is a potent antifungal drug. The addition of the tablet was intended to inhibit the growth of bacteria and fungi in the milk while preserving the existing microorganisms. Moreover, it enabled the milk to stabilize during transport. A total of 55 samples of milk were analyzed. The samples were collected in sterile containers and transported to the microbiological laboratory at 4 °C, and then stored at −20 °C.

### 4.3. Isolation of DNA

DNA isolation from milk samples was performed using a Genomic Mini AX Bacteria Spin Kit (A&A Biotechnology, Gdańsk, Poland) according to the protocol provided by the manufacturer. The method was based on the binding of DNA to a silica membrane. First, microbial cells were lysed with the use of the enzyme lysozyme and a buffer containing chaotropic salts that inactivate nuclease. Protein impurities were removed by enzymatic hydrolysis with proteinase K. After centrifugation of the remaining cell biomass, the supernatant was loaded onto columns with silica membranes. The systems were washed twice with low ionic strength buffers. Finally, the purified DNA was washed away. The isolates were stored at −80 °C, after they had been neutralized, in order to minimize matrix degradation.

The efficiency of isolation was checked each time based on the fluorometric method with the use of a Qbit 3.0 device and a Qubit™ dsDNA HS Assay Kit (ThermoFisher Scientific, Carlsbad, CA, USA). The purity and integrity of the extracted DNA were determined by spectrophotometric analysis with a Nanodrop 2000 (Thermo Fisher Scientific, Waltham, MA, USA) and agarose gel electrophoresis, respectively. For each sample, three DNA extractions were performed and finally combined after a positive quantification.

### 4.4. PCR Amplification and NGS Sequencing

The PCR reaction was prepared using an Ion 16S™ Metagenomics Kit (Life Technologies, Waltham, MA, USA). This kit allowed for the amplification of the V2–V9 regions of the bacterial 16S rRNA gene. The reaction was prepared according to manufacturer’s instructions. The reaction mixture consisted of 15 µL of Environmental Master Mix, 3 µL of the appropriate primer, and 12 µL of DNA sample previously isolated from the milk sample. The reaction was performed in a Veriti thermal cycler (Life Technologies, Carlsbad, CA, USA) using the following temperature program: initial denaturation at 95 °C for 10 min; 25 cycles of denaturation for 30 s at 95 °C, annealing for 30 s at 58 °C, extension for 20 s at 72 °C, and a final extension at 72 °C for 7 min.

The reaction products were purified using the Agencourt AMPure XP Reagent (Bentley Instruments, Chaska, MN, USA) according to the manufacturer’s instructions. The method was based on binding DNA to magnetic beads, followed by washing away the contaminants with ethanol. DNA was rinsed from the beads using nuclease-free water or low-TE buffer. A library was prepared according to the manufacturer’s instructions using an Ion Plus Fragment Library Kit (Life Technologies, Carlsbad, CA, USA). The prepared library was purified using Agencourt AMPure XP Reagent (Beckman Coulter), according to the manufacturer’s instructions. The concentration of the library was assessed using an Ion Universal Library Quantitation Kit and a real-time PCR instrument—Quant Studio 5 (Life Technologies, Carlsbad, CA, USA). The library was then diluted to a concentration of 10 pM. The diluted library was coated onto beads (used for sequencing) in emulsion PCR using an Ion PGM™ Hi-Q™ View OT2 Kit reagent Kit and an Ion One Touch 2 Instrument (Life Technologies, Carlsbad, CA, USA). The library coated beads were purified using an Ion One Touch ES Instrument (Life Technologies, Carlsbad, CA, USA). The bead-coated library prepared this way was sequenced using an Ion PGM System (Life Technologies, Carlsbad, CA, USA) with an Ion PGM™ Hi-Q™ View Sequencing Kit on an Ion 316™ Chip Kit v2 BC.

### 4.5. Bioinformatic Analysis

The sequence reads from the Ion Torrent (Thermo Fisher Scientific, Waltham, MA) in BAM format were imported into CLC Genomics Workbench 20.0 software and processed with CLC Microbial Genomics Module 20.1.1 (Qiagen, Denmark). The total number of reads and the results of downstream processing for all samples are presented in the Supplementary Materials (Table S2). Chimeric and low-quality reads (quality limit = 0.05, ambiguous limit = ‘N’) were filtered and removed. Then, the sequence reads were clustered against the SILVA v119 [30] database at 97% similarity of operational taxonomic units (OTUs). Finally, a merged abundance table was generated, and the selected alpha (number of OTUs, Chao-1 bias-corrected, Shannon entropy, and Phylogenetic diversity) and beta (Bray–Curtis principal coordinate analysis) diversity parameters were determined.

### 4.6. Determination of Pathogens

The PCR reaction was prepared with a ready-made polymerase mixture, along with all the necessary components to perform a PCR reaction using a TaqMan™ Fast Advanced Master Mix Kit (Life Technologies, Carlsbad, CA, USA). A list of primers used in the qPCR reaction is provided in Table 3. The composition of the reaction mixture was as follows. The reaction was prepared according to the following scheme: Fast Advanced Master Mix, 12.5 µL; H_2_O, 2.25 µL; L_MO_F, 0.1 µL (0.4 µM); L_MO_R, 0.1 µL (0.4 µM); L_MO_P, 0.05 µL (0.2 µM); DNA (or positive or negative control), 10 µL. The reaction was carried out using the following temperature program: Initial denaturation at 95 °C for 2 min and 45 cycles, denaturation at 95 °C for 15 s, and annealing and elongation at 55 °C for 1 min. All reactions were performed using a QuantStudio 5 apparatus (ThermoFisher, Waltham, MA, USA). In order to verify the operation of the developed method, certified standards from Vircell and Identifica (Table S3) were used (Figures S1 and S2, Table S3)

### 4.7. Determination of the Number of Somatic Cells in Milk

Milk samples for SCC determination were analyzed using Fossomatic 90 (Foss Electric, Hillerød, Denmark).

## 5. Conclusions

This study demonstrated the complementarity of culture-independent methods for evaluating the bacterial populations that may be present in raw milk. Studies profiling the bacterial communities of raw milk can demonstrate changes in the milk microbiome depending on the health of the cows. The presence of pathogens, including *Escherichia coli* and *S. agalactiae*, is a serious public and animal health problem, as they may enter the dairy products consumed by humans.

## Figures and Tables

**Table 1:** Basic characteristics and classifications of the milk samples (coded) included in this study.

Milk Samples	Identified Bacteria	SSC ^1^ < 225,000	225,000 < SSC < 400,000
*S. agalactiae subclinical mastitis milk* (M)	*Streptococcus agalactiae*	Negative	Positive (M1–M15)
*E. coli subclinical mastitis milk* (E)	*Escherichia coli*	Negative	Positive (E1–E16)
*Healthy milk* (H)	-	H1–H24	-

^1^ Cells/mL.

**Table 2:** Analysis of the alpha-biodiversity of the bacteria present in the analyzed milk samples.

	Healthy Milk (*p* < 0.0001, *F* = 516.3)	*E. coli* Subclinical Mastitis Milk (E) (*p* < 0.0001, *F* = 314.7)	*S. agalactiae* Subclinical Mastitis Milk (M) (*p* < 0.0001, *F* = 411.5)
OUT number	5319 ± 96	4239 ± 102	3987 ± 156
Chao 1	5498 ± 82	4519 ± 52	4019 ± 69
Shannon index	7.98 ± 0.54	6.81 ± 0.19	6.51 ± 0.38
Phylogenetic diversity	10.11 ± 0.52	8.54 ± 0.39	7.85 ± 0.68

Also shown is the significance between the parameters determined by ANOVA (*p*, *F*).

**Table 3:** Primers and probes for quantitative real time PCR.

Microorganism	Gene	Sequences of Primers and Probes	
*E. coli*	uidA	Forward primer (5′–3′) Reverse primer (5′–3′) Probe	GTGTGATATCTACCCGCTTCGC AGAACGGTTTGTGGTTAATCAGGA TCGGCATCCGGTCAGTGGCAGT
*Streptococcus agalactiae*	cfb	Forward primer (5′–3′) Reverse primer (5′–3′) Probe	AGCTGTATTAGAAGTACATGCT CATTTGCTGGGCTTGATTATT ATCAAGTGACAACTCCACAAGTGGTAA
*Bos taurus* (control)	*mtDNA*	Forward primer (5′–3′) Reverse primer (5′–3′) Probe	CGGAGTAATCCTTCTGCTCACAGT GGATTGCTGATAAGAGGTTGGTG CATGAGGACAAATATC

## Supplementary Materials

The following supporting information can be downloaded at: https://www.mdpi.com/article/10.3390/molecules27061829/s1, Figure S1: Construction of the standard curves for *S. agalactiae* detection; Figure S2: Construction of the standard curves for *E. coli* detection; Table S1: The relative abundance of bacterial genera (%) in milk from healthy cows and cows with subclinical mastitis; Table S2: The results of read-filtering in sequencing data analysis; Table S3: Certified standards of bacterial genomes from Vircell and Identifica.
