# Supplementary material for: The Influence of Bacteria Causing Subclinical Mastitis on the Structure of the Cow’s Milk Microbiome
Source: Molecules. 2022 Mar 11;27(6):1829. doi: 10.3390/molecules27061829 (PMC8950352; doi:10.3390/molecules27061829)
Supplement: Supplementary file 1 [file molecules-27-01829-s001.zip › molecules-1611297-supplementary.pdf]

Supplementary materials

Table S1. The relative abundance of bacterial genera (%) in milk from healthy cows and cows with sub-clinical mastitis.

**Genious**	**Healthy**	**S. agalactiae-submastitis milk**	**E. coli-submastitis milk**
Bacillus	15.0 ± 5.6	3.5 ± 2.1	9.5 ± 3.7
Escherichia-Shigella	4.6 ± 1.9	2.1 ± 1.6	24.8 ± 6.9
Streptococcus	3.3 ± 2.1	25.1± 7.1	4.1 ± 2.9
Pseudomonas	10. 3 ± 4.8	2.1 ± 0.9	5.7 ± 2.8
Corynebacterium	4.7 ± 1.6	4.6 ± 3.4	8.2 ± 3.3
Acinetobacter	2.9 ± 0.9	4.4 ± 2.2	3.0 ± 1.9
Aeromonas	0.2 ± 0.2	9.1 ± 4.6	0.2 ± 0.2
Chryseobacterium	0.3 ± 0.2	6.9 ± 3.1	1.0 ± 0.6
LAB	12. 3 ± 4.1	3.1 ± 1.8	5.0 ± 1.9
Turicibacter	2.3 ± 0.9	4.0 ± 2.7	0.8 ± 0.6
Bacteroides	3.8 ± 2.6	0.7 ± 0.6	1.2 ± 0.7
Staphylococcus	4.7 ± 3.4	4.5 ± 2.6	4.0 ± 2.4
Aerococcus	2.4 ± 1.6	2.6 ± 1.8	1.0 ± 0.8
Dyella	0.3 ± 0.2	1.1 ± 0.8	0.0
Fusobacterium	1.9 ± 1.1	0.0	0.1 ± 0.1
Stenotrophomonas	0.3 ± 0.3	0.4 ± 0.3	2.4 ± 1.6
{Unknown Phylum} Bacteria	2.6 ± 1.6	5.0 ± 2.6	1.8 ± 1.2
Flavobacterium	0.2 ± 0.1	0.6 ± 0.3	1.8 ± 0.9
Salmonella	3.6 ± 1.9	0.4 ± 0.3	3.3 ± 2.6
Acidovorax	0.1 ± 0.1	3.4 ± 1.8	0.9 ± 0.6.
Ignavigranum	1.3 ± 1.2	2.3 ± 1.7	0.3 ± 0.2
uncultured-069	4.2 ± 3.5.	3.2 ± 2.4	1.4 ± 0.6
uncultured Fusobacterium sp.	1.3 ± 0.9	0.0	0.0
Enterobacter	4.8 ± 2,6	1.1 ± 0.6	5.7 ± 3.4
Facklamia	1.5 ± 0.9	0.1± 0.1	0.2 ± 0.2
Variovorax	2.6 ± 1.5	0.2± 0.2	0.2 ± 0.1
Oligella	0.2 ± 0.2	0.4± 0.3	1.0 ± 0.8
Jeotgalicoccus	0.2 ± 0.1	1.2 ± 0.6	0.4 ± 0.3
Globicatella	0.2 ± 0.1	1.0 ± 0.7	0.1 ± 0.1
Helcococcus	0.8 ± 0.6	0.6 ± 0.5	0.2 ± 0.1
Microvirgula	0.9 ± 0.4	0.0 ± 0.6	0.9 ± 0.6
uncultured-053	1.4 ± 1.1	1.5 ± 1.2	0.5 ± 0.4
Sphingobacterium	0.1 ± 0.1	0.2 ± 0.1	1.3 ± 0.6
uncultured marine bacterium-009	1.0 ± 0.6	0.1 ± 0.1	2.6 ± 1.3
Rahnella	0.6 ± 0.5	0.0	0.0
uncultured-100	0.0	0.6 ± 0.4	0.1 ± 0.1

uncultured-107	0.0	0.8 ± 0.5	0.1 ± 0.1
Delftia	0.0	0.0	0.6 ± 0.4
Bifidobacterium	1.0 ± 0.6	0.3 ± 0.2	0.4 ± 0.3
Butyrivibrio	1.0 ± 0.7	1.0 ± 0.6	1.1 ± 1.0
Rhodanobacter	0.0	0.2 ± 0.2	0.0
Veillonella	0.4 ± 0.3	0.1 ± 0.1	2.0 ± 1.2
Methylophilus	0,2 ± 0.2	0.0	0.6 ± 0.4
Dietzia	0.3 ± 0.2	0.4 ± 0.2	0.3 ± 0.3
Brevundimonas	0.1 ± 0.1	0.0	0.9 ± 0.4
Sideroxydans	0,0	0.2 ± 0.1	0.0
uncultured bacterium-360	0,0	0.7 ± 0.5	0.0
uncultured-104	0,0	0.0	0.4 ± 0.3

Table S2. The results of read-filtering in sequencing data analysis.

**Sample name**	**Number of reads**	**Filtered reads**	**Unique reads after filtering**	**Chimeric reads**	**Unique chimeric reads**	**Reads in OTUs**
H1	185551	64634	21716	11252	917	109666
H2	37142	15259	4741	964	132	20919
H3	64833	32272	8993	1684	327	30877
H4	62505	20742	8260	4378	394	37386
H5	25410	10734	3222	702	95	13974
H6	8065	3820	826	18	8	4231
H7	179794	50685	21021	13881	594	115228
H8	95898	43542	11660	1632	268	50723
H9	276311	99081	34460	13531	1231	163699
H10	275366	105430	34731	9711	1100	160226
H11	560894	105424	83652	26458	2793	429012
H12	347007	105910	55459	13894	3102	227203
H13	121930	32658	20323	4502	1011	75676
H14	454938	132605	71515	26587	5068	295746
H15	432541	100594	68589	30454	5299	301494
H16	579901	106458	89449	30293	3421	443150

H17	696252	124256	106313	32902	4289	539094
H18	484128	106171	76617	19856	3502	358101
H19	392135	86137	60163	16966	2384	289032
H20	491930	99882	76425	28019	3440	364029
H21	285117	55971	26827	38276	1261	151270
H22	264217	65342	24487	32479	825	164640
H23	230086	49871	21517	23470	703	156746
H24	276177	61986	25186	38303	980	175888
E1	279164	55648	25477	50255	1213	173261
E2	312626	78793	30677	36660	1310	197173
E3	224370	72829	23476	12571	673	138971
E4	244317	76694	24654	20188	870	147434
E5	301037	94940	32416	26616	1705	179482
E6	224287	75125	23261	17575	744	131588
E7	134946	47006	15794	8183	667	79757
E8	27012	11098	3448	701	96	15214
E9	47151	23470	6540	1225	238	22456
E10	45458	15085	6007	3184	286	27190
E11	18480	7806	2343	510	69	10163
E12	5866	2778	601	13	6	3077
E13	130759	36862	15288	10095	432	83802
E14	69744	31667	8480	1187	195	36890
E15	200954	72059	25062	9841	895	119054
E16	200266	76676	25259	7062	800	116528
M1	407923	76672	60838	19242	2031	312009
M2	252369	77026	40334	10105	2256	165238
M3	88676	30365	14780	3274	735	55037
M4	330864	96440	52011	19336	3686	215088
M5	314575	73159	49883	22148	3854	219268
M6	421746	77424	65054	22031	2488	322291

M7	506365	90368	77318	23929	3119	392068
M8	352093	77215	55722	14441	2547	260437
M9	285189	62645	43755	12339	1734	210205
M10	357767	72642	55582	20378	2502	264748
M11	207358	40706	19510	27837	917	110014
M12	190881	47522	17809	23621	600	119738
M13	167335	36270	15649	17069	511	113997
M14	200856	45081	18317	27857	713	127918
M15	203028	40471	18529	36549	882	126008

Table S3. Certified standards of bacterial genomes from Vircell and Identifica.

Microorganism	Catalog number	Concentration	Producer
E.coli	GDNA0005	50 ng/μl	IDENTIFICA
Streptococcus agalactiae	MBC071	10 000 copy/μl	Vircell
Bos taurus (control)	MBC124	10 000 copy/μl	Vircell

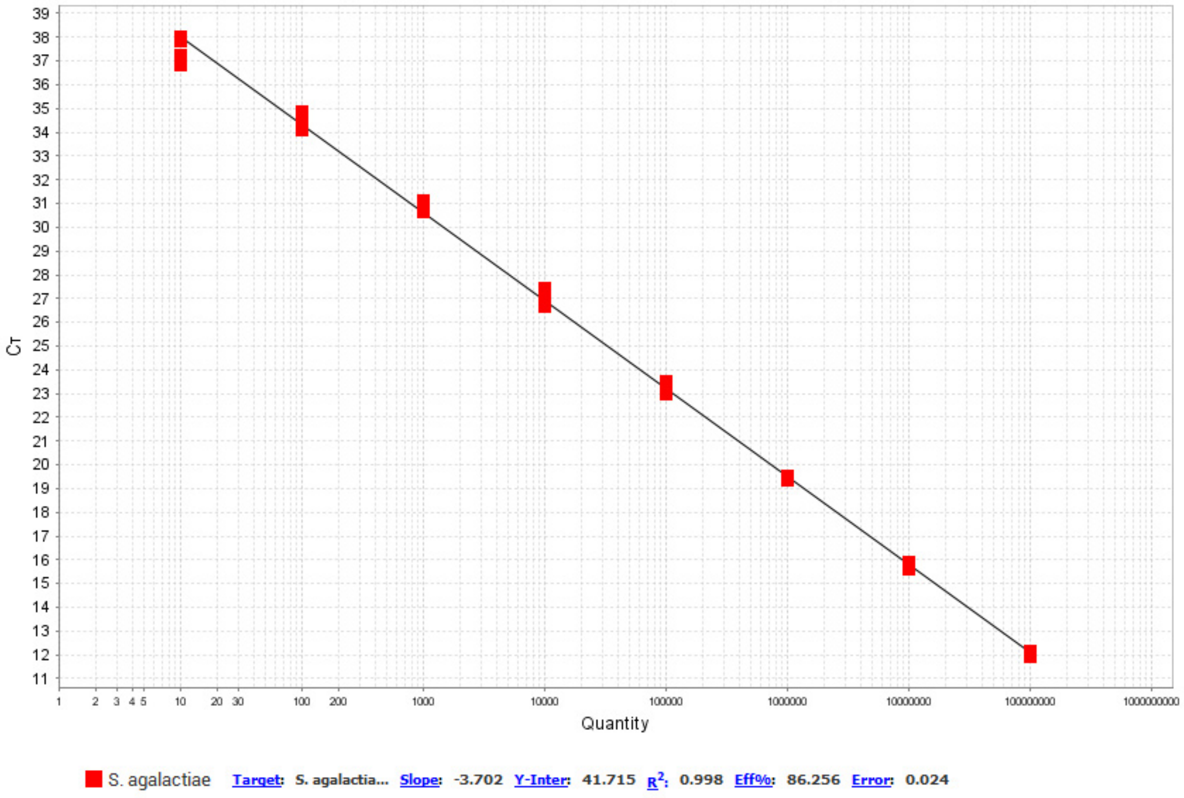

Figure S1. Construction of the standard curves for *S. agalactiae* detection.

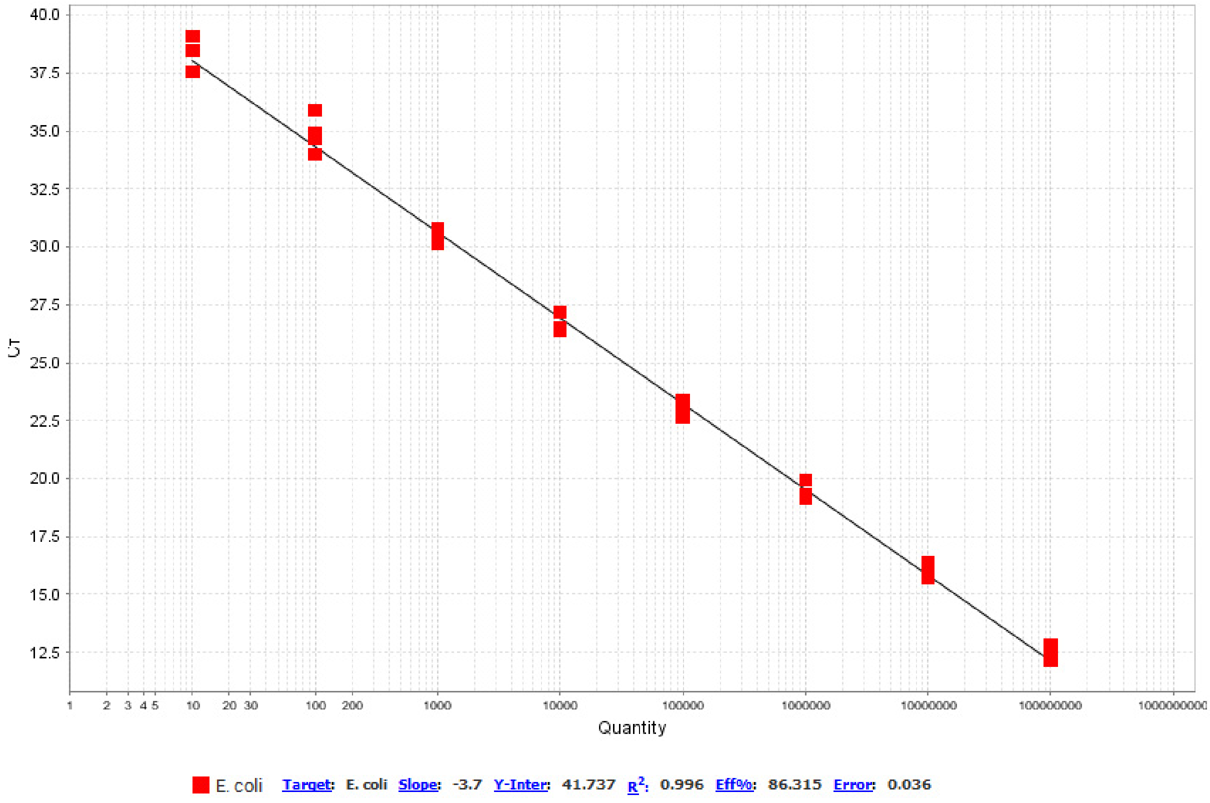

Figure S2. Construction of the standard curves for *E. coli*. Detection.
